# The Multi-Functional Calcium/Calmodulin Stimulated Protein Kinase (CaMK) Family: Emerging Targets for Anti-Cancer Therapeutic Intervention

**DOI:** 10.3390/ph12010008

**Published:** 2019-01-07

**Authors:** Joshua S. Brzozowski, Kathryn A. Skelding

**Affiliations:** Priority Research Centre for Cancer Research, Innovation and Translation, School of Biomedical Sciences and Pharmacy, Hunter Medical Research Institute (HMRI) and University of Newcastle, Callaghan, NSW 2308, Australia; joshua.brzozowski@newcastle.edu.au

**Keywords:** CaMKK, CaMKI, CaMKII, CaMKIV, anti-cancer drugs

## Abstract

The importance of Ca^2+^ signalling in key events of cancer cell function and tumour progression, such as proliferation, migration, invasion and survival, has recently begun to be appreciated. Many cellular Ca^2+^-stimulated signalling cascades utilise the intermediate, calmodulin (CaM). The Ca^2+^/CaM complex binds and activates a variety of enzymes, including members of the multifunctional Ca^2+^/calmodulin-stimulated protein kinase (CaMK) family. These enzymes control a broad range of cancer-related functions in a multitude of tumour types. Herein, we explore the cancer-related functions of these kinases and discuss their potential as targets for therapeutic intervention.

## 1. Introduction

Ca^2+^ is a major second messenger in cells and is essential for a variety of important signalling pathways. Alterations in intracellular Ca^2+^ signalling regulate many biological processes, including gene transcription, exocytosis, the cell cycle, migration and apoptosis. Cytoplasmic Ca^2+^ concentrations rise in response to a variety of stimuli, which activate Ca^2+^-channels in the plasma membrane, or by release from intracellular stores. It is increasingly being realised that disruption of normal Ca^2+^ signalling contributes to the development of tumourigenic phenotypes [1], and aberrant Ca^2+^ signalling has been implicated in each of the hallmarks of cancer originally identified by Hanahan and Weinberg [2].

Ca^2+^ signals in the form of spikes or oscillations and is tightly regulated. The decoding of this is achieved by several downstream effectors, including calmodulin (CaM). Binding of Ca^2+^ dramatically changes the conformation of CaM and increases its affinity for a large number of CaM-binding proteins, including the multifunctional CaM kinases (CaMKK, CaMKI, CaMKII and CaMKIV). These multifunctional kinases are widely expressed and control a variety of cancer related functions in a range of cancer types. Their potential as targets for anti-cancer therapeutic intervention have recently begun to be appreciated.

## 2. Structure and Regulation of Calcium/Calmodulin-Stimulated Protein Kinase (CAMK) Family Members

### 2.1. CaMKK

Ca^2+^/calmodulin stimulated protein kinase kinase (CaMKK) is a multifunctional protein kinase encoded by two genes (*CAMKK1* and *CAMKK2*) that produce CaMKKα or CaMKKβ [3], respectively. The *CAMKK2* gene produces several splicing isoforms depending on cell type [4,5]. CaMKKα was originally identified in rat brain as an activating kinase for CaMKIV [6] and CaMKI [7], and an additional β isoform was later identified [8]. CaMKK is primarily expressed in the brain, including the olfactory bulb, hypothalamus, hippocampus, dentate gyrus, cerebellum and amygdala, and at low levels in peripheral tissues (such as the thymus, spleen, lung, and testis) [9,10,11]. 

CaMKK phosphorylates CaMKI and CaMKIV [3,11], AMP activated protein kinase (AMPK) [12] and protein kinase B (PKB/Akt) [13]. CaMKK, CaMKI and CaMKIV form a signalling pathway termed the Ca^2+^/CaM-dependent kinase cascade, which has been implicated in several cellular processes, including regulating dendritic spine morphology, hematopoietic stem cell maintenance, cell proliferation, apoptosis, glucose uptake, adipogenesis, and normal immune cell function [14,15,16,17,18,19,20,21]. 

**Figure 1:**
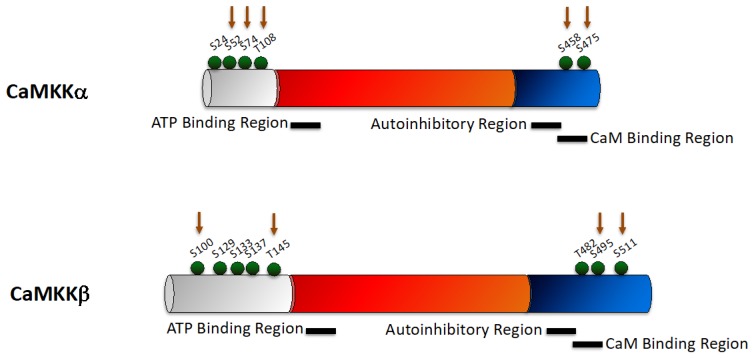
Schematic representing the domain structure of CaMKK. There are two CaMKK isoforms—CaMKKα and CaMKKβ. CaMKK consists of a unique N-terminal domain (grey), a catalytic domain (red) which contains an ATP binding region, and a regulatory domain (blue) containing overlapping autoinhibitory and calmodulin (CaM) binding regions. Phosphorylation sites are indicated by green balls, with protein kinase A (PKA) phosphorylation sites indicated with red arrows.

Although CaMKKα and CaMKKβ share high sequence homology (Figure 1) and possess a common domain structure, with a catalytic domain adjacent to an autoregulatory domain containing an autoinhibitory region that overlaps with the CaM-binding region, they differ in their biochemical properties. Whilst CaMKKα requires Ca^2+^/CaM to relieve the autoinhibitory mechanism [22], CaMKKβ exhibits partially autonomous activity in the absence of Ca^2+^/CaM [8,11], which is regulated by phosphorylation by glycogen synthase kinase 3β (GSK3β) and cyclin-dependent kinase 5 (CDK5) [23]. CDK5 phosphorylates CaMKKβ at S137, thereby priming CaMKKβ for phosphorylation by GSK-3β at S129 and S133 [23]. Both CaMKK isoforms are partly inhibited following cAMP-dependent protein kinase (PKA) phosphorylation [24,25], and PKA can phosphorylate CaMKKα on S52, S74, T108, S458, and S475 [25], and CaMKKβ on S100, S495 and S511 [10]. The major site of autophosphorylation of CaMKKα is S24 [25], and for CaMKKβ is T482 [26]. T482 generates partial autonomous activity, which results in partial disruption of the autoinhibitory mechanism [26]. As CaMKKβ is not dependent on rapid fluxes in intracellular Ca^2+^ for basal activity, it can respond to other stimuli of longer duration and can phosphorylate AMPK.

Binding of Ca^2+^/CaM to CaMKKα prevents phosphorylation at S52, S74, T108, and S458, but enhances phosphorylation at S475 [25]. Additionally, phosphorylation of CaMKKα on S74, T108 and S458 negatively regulates activity [24,27,28] and phosphorylation of S458 blocks Ca^2+^/CaM binding. 

### 2.2. CaMKI

The Ca^2+^/calmodulin-stimulated protein kinase I (CaMKI) family is composed of four members, which are coded for by four different genes: *CAMK1*, *PNCK*, *CAMK1G* and *CAMK1D*, which produce CaMKIα, CaMKIβ/Pnck, CaMKIγ/CLICK3, or CaMKIδ/CKLiK, respectively. The *PNCK* gene produces several splicing isoforms depending on cell type and developmental stage [29]. The various isoforms of CaMKI are ubiquitously expressed at low levels [30], and expressed at high levels in several brain regions, including the cortex, hippocampus, thalamus, hypothalamus, midbrain, and olfactory bulb, with each isoform exhibiting distinct spatiotemporal expression during neuronal development [31,32]. CaMKI has been implicated in the control of a variety of cellular functions, including long term potentiation, the control of synapsin in nerve terminals, axon/dendritic outgrowth and growth cone motility, aldosterone synthase expression, osteoclast differentiation and bone resorption and proliferation [32,33,34,35,36,37,38,39].

**Figure 2:**
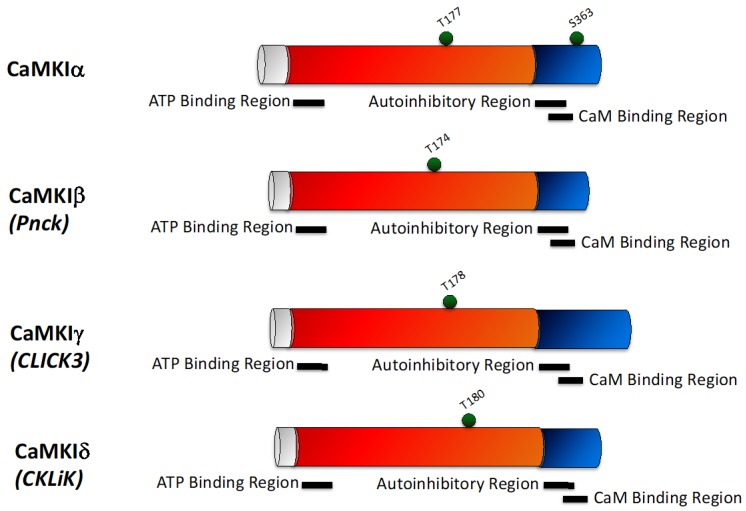
Schematic representing the domain structure of the CaMKI family. There are four CaMKI isoforms—CaMKIα, CaMKIβ, CaMKIγ, and CaMKIδ, with each isoform sharing a similar structure. CaMKI consists of a unique N-terminal domain (grey), adjacent to a catalytic domain (red) which contains an ATP binding region, and a regulatory domain (blue) containing overlapping autoinhibitory and calmodulin (CaM) binding regions. Phosphorylation sites are indicated by green balls.

Similar to CaMKK, the four CaMKI isoforms share a common domain structure (Figure 2), with a catalytic domain adjacent to an autoregulatory domain containing an autoinhibitory region that overlaps with the CaM-binding region. Binding of Ca^2+^/CaM to CaMKI causes a conformational change that relieves the autoinhibition and allows phosphorylation by CaMKK (T174 for CaMKIα), which is required for maximal CaMKI activity [40,41]. Additionally, once CaMKIδ is phosphorylated by CaMKK, it becomes resistant to protein phosphatases, which induces a ‘primed’ state, where it can more readily be activated in response to Ca^2+^ signals than other CaMKI enzymes [42]. 

### 2.3. CaMKII

The Ca^2+^/calmodulin-stimulated protein kinase II (CaMKII) family is encoded by four genes: *CAMK2A*, *CAMK2B*, *CAMK2G*, *CAMK2D*, which produce CaMKIIα, CaMKIIβ, CaMKIIγ, and CaMKIIδ, respectively. Alternative splicing within the variable linker-region produces multiple isoforms [43]. One or more members of the CaMKII family are found in every tissue. CaMKIIα and CaMKIIβ are most highly expressed in neurons and are involved in regulating a variety of neuronal functions, including neurotransmitter synthesis and release, neurite extension, and synaptic plasticity that underlies learning and memory [44,45,46,47,48]. CaMKII has also been implicated in the regulation of non-neuronal processes, including fertilisation, the maintenance of vascular tone, osteogenic differentiation, normal cardiac function, apoptosis and excitotoxicity/ischaemic-induced cell death and cell proliferation [34,49,50,51,52,53,54,55,56]. 

**Figure 3:**
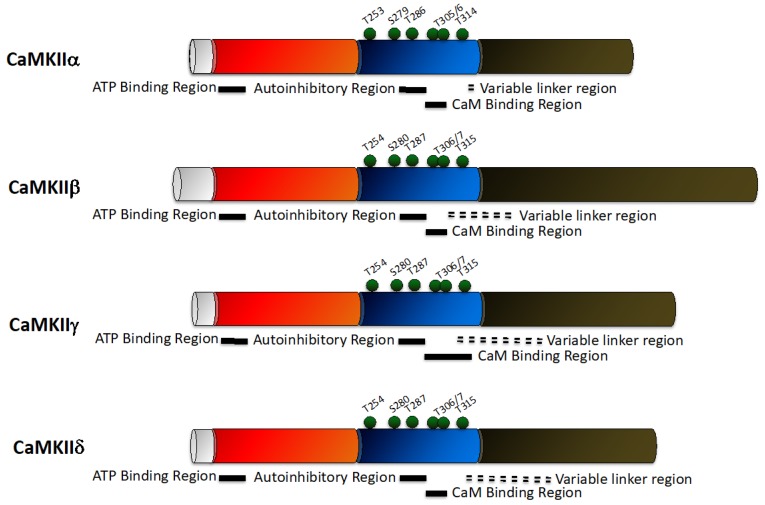
Schematic representing the domain structure of the CaMKII family. There are four CaMKII isoforms—CaMKIIα, CaMKIIβ, CaMKIIγ, and CaMKIIδ, with each isoform sharing a similar structure. CaMKII consists of a unique N-terminal domain (grey), adjacent to a catalytic domain (red) which contains an ATP binding region, and a regulatory domain (blue) containing overlapping autoinhibitory and calmodulin (CaM) binding regions. Phosphorylation sites are indicated by green balls. All isoforms also contain a C-terminal association domain (brown), which is involved in the formation of CaMKII multimers.

Similar to the other CaMK family members, CaMKII subunits (Figure 3) have an N-terminal catalytic domain, and a regulatory domain (which contains autoinhibitory and CaM binding domains). In contrast to the other CaMK family members, CaMKII possesses a C-terminal association domain and associates into a multimeric form [57]. The crystalline structure of CaMKIIα shows that it consists of two autoinhibited catalytic domains in a symmetric dimer. The regulatory domain is joined to the C-terminus of the catalytic domain [57], which functions as a gate (with T286 as its hinge), so that it blocks the protein substrate and adenosine triphosphate (ATP) binding sites when CaMKII is autoinhibited and is ‘open’ following autophosphorylation at T286. CaMKII is therefore comprised of six mutually inhibited dimers. 

CaMKII requires Ca^2+^/CaM for initial enzyme activity. Binding of CaM molecules to adjacent subunits within a holoenzyme allows autophosphorylation at T826 of one or both of these subunits to occur [58]. Autophosphorylation of T286 in CaMKIIα (T287 in CaMKIIβ, γ, and δ) occurs rapidly and alters the affinity for Ca^2+^/CaM and enzyme activity [59,60]. CaMKII phosphorylation at T286 generates an autonomously active kinase that remains active even following CaM dissociation. Phosphorylation at T286 can also regulate the function of CaMKII by altering its binding to specific subcellular locations [61,62,63]. Once CaMKII is Ca^2+^-independent, and Ca^2+^/CaM is no longer bound, secondary sites within the CaM-binding site can be phosphorylated (T305/306 in CaMKIIα, and T306/307 in CaMKIIβ, γ, and δ) [64,65]. Phosphorylation at these sites prevents CaM binding and CaMKII subsequently becomes insensitive to changes in Ca^2+^/CaM [66]. 

Other forms of post-translational modification have also been demonstrated to alter CaMKII activity. Specifically, a pair of methionine residues (M281/282), present in the β, γ, and δ, but not α, isoforms [67], can become oxidised and produce a conformational change in CaMKII, similar to that produced following T286 phosphorylation, leading to an autonomous activation of CaMKII [68]. However, not all CaMKII phosphorylation sites modulate Ca^2+^/CaM binding and kinase activity. Phosphorylation at several sites that have no direct effect on kinase activity, but alter molecular targeting, have been identified in vivo, including T253, S279 and S314 [69,70,71,72]. 

### 2.4. CaMKIV

Ca^2+^/calmodulin-stimulated protein kinase IV (CaMKIV) is encoded by the *CAMK4* gene. Two different isoforms (CaMKIVα and CaMKIVβ) are produced by alternative processing [73,74]. The CaMKIV expression pattern is similar to that of CaMKKβ, with both primarily being expressed in the brain, however, CaMKIV is also present in immune cells, the testes and ovaries [9,16,75,76]. It is particularly enriched in cerebellar granule cells, and subsequently, has previously been referred to as CaMK-Gr. The *CAMK4* gene also encodes calspermin, a Ca^2+^/CaM binding protein of unknown function that is exclusively expressed in spermatids in the testes [76]. CaMKIV has been implicated in the regulation of homeostatic plasticity, neurite outgrowth, fear memory, immune and inflammatory responses, the regulation of cyclic AMP element binding protein (CREB) and cell proliferation [34,77,78,79,80,81].

**Figure 4:**
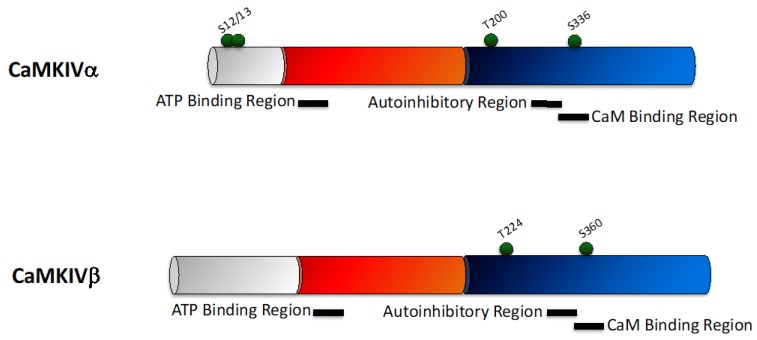
Schematic representing the domain structure of the CaMKIV family. The two CaMKIV isoforms—CaMKIVα and CaMKIIβ differ only at their N-terminus. CaMKIV consists of a unique N-terminal domain (grey), adjacent to a catalytic domain (red) which contains an ATP binding region, and a regulatory domain (blue) containing overlapping autoinhibitory and calmodulin (CaM) binding regions. Phosphorylation sites are indicated (green balls).

CaMKIVβ is identical to CaMKIVα, except for the addition of 28 amino acids at the N-terminus. Similar to the other CaMK family members, CaMKIV has a catalytic domain adjacent to an autoregulatory domain containing an autoinhibitory region that overlaps with the CaM-binding region (Figure 4). CaMKIV requires Ca^2+^/CaM to become active, as well as phosphorylation of the conserved T in the activation loop (T200 in human CaMKIV and T196 in the rat enzyme) by CaMKK [82], which generates an autonomously active kinase. Following this, CaMKIV then autophosphorylates at S12 and S13, which enable Ca^2+^/CaM independent activity. Double S11A/S12A phospho-null mutants lack Ca^2+^/CaM-dependent basal activity and are unresponsive to CaMKK [83], indicating that S12 and S13 mediate an intrasteric inhibition, and are essential for full activation of CaMKIV. Similar to T305/6 in CaMKII, phosphorylation of CaMKIV within the CaM binding region (S336 in human CaMKIV and S332 in the rat enzyme) prevents CaM binding and inactivates CaMKIV [84].

## 3. The Role of CaMK Family Members in Cancer

Due to their role as mediators of Ca^2+^-signalling, it is not surprising that the CaMK family members have been identified as being overexpressed or aberrantly activated in a wide variety of cancer types. Additionally, each have also been implicated in controlling cellular processes important in cancer-related functions, such as cell proliferation, apoptosis, motility and invasion. 

### 3.1. CaMKK

Despite the restricted expression of CaMKKβ in normal cells, it is overexpressed in several types of cancer compared to adjacent benign tissue (Table 1), including gastric tumours [85], hepatocellular carcinoma [86], high-grade gliomas [87], ovarian [88] and prostate cancer [89,90,91]. Androgen stimulation of androgen-dependent and castration-resistant prostate cancer cell lines results in upregulation of CaMKKβ [89,91,92], indicating direct regulation by the androgen receptor. Subsequent studies have shown that CaMKKβ is a key effector of the androgen receptor in stimulating glycolysis through the activation of AMPK and phosphofructokinase (PFK), which drives anabolism and proliferation of prostate cancer cells [91]. Furthermore, CaMKKβ expression increases with human prostate cancer progression and Gleason score [89,91], and with tumour progression in a transgenic adenocarcinoma of the mouse prostate (TRAMP) mouse model of prostate cancer [89], suggesting that it may also play a role in prostate cancer progression. 

Increased CaMKKβ expression also correlates with poor patient survival in additional tumour types (Table 1). High CaMKKβ expression is associated with poor disease-free survival in hepatic cancer patients [86], and poor overall survival in glioma patients [87]. These studies suggest that CaMKKβ may be a useful prognostic marker for liver and brain cancers.

CaMKKβ positively regulates cancer cell proliferation, migration and invasion in a variety of cell types in vitro (Table 2). Downregulation of CaMKKβ expression using small interfering RNA (siRNA) or pharmacological inhibition inhibits prostate cancer cell proliferation [89,91,92,107], migration and invasion [92]. Conversely, CaMKKβ overexpression in LNCaP prostate cancer cells increases cell migration, further supporting a role for CaMKKβ overexpression in prostate cancer progression [92]. By contrast, Shima et al. showed that CaMKKβ overexpression in LNCaP cells decreases cell proliferation and tumour growth in vivo [90], indicating that further examination of the role of CaMKKβ in prostate cancer cell proliferation is required. Downregulation or pharmacological inhibition of CaMKKβ also decreases proliferation, migration and invasion of glioma [87], gastric [85,108] and liver cancer [86] cells and expression of a dominant negative CaMKK mutant suppresses medulloblastoma cell migration [109], suggesting that CaMKK activity is essential for this process. Taken together, these studies highlight the importance of CaMKK in controlling cancer cell proliferation and metastatic processes in a range of cancer types, indicating that its role in these functions is not cell-type specific, and also suggesting that CaMKKβ may be a valid anti-cancer target for a variety of cancer types.

### 3.2. CaMKI

The various CaMKI isoforms have been shown to be overexpressed in several different cancer subtypes (Table 1). CaMKI is more highly expressed in stage III and IV endometrial carcinomas, when compared to stage I and II, and is associated with proliferating cellular nuclear antigen (PCNA)-labelling, clinical state, histological grade, and the presence of invasion, indicating that CaMKI may play a role in endometrial carcinoma progression [94]. Expression of several of the specific CaMKI isoforms have also been examined in specific cancer types. For example, CaMKIγ/PNCK is overexpressed in a subset of primary breast cancers compared to benign mammary tissue [95], and in clear renal cell carcinoma compared to adjacent non-tumour tissues [96]. Furthermore, a significant correlation between PNCK expression and Fuhrman grade, tumour size, T and N stage was observed in these clear cell renal carcinoma samples, and high levels of PNCK is an independent predictor for poor patient survival [96]. *CAMKID* has also been identified as a potential prognostic marker in acute myeloid leukaemia (AML), as high levels of *CAMKID* mRNA are associated with significantly worse AML patient survival [93]. 

Unsurprisingly, CaMKI has been implicated in a variety of cancer-related cellular processes (Table 2). CaMKI controls the progression of MCF-7 breast cancer of cells through G_1_ [110], potentially by regulating cdk4 and retinoblastoma protein (Rb) phosphorylation, as overexpression of the kinase-inactive CaMKI K49A mutant prevents cdk4 activation and Rb hyperphosphorylation in WI-38 fibroblasts [17]. Indeed, knockdown of CaMKI expression in MV-4-11 and Kasumi-1 AML cells significantly decreases cell proliferation and inhibits leukaemia development and prolongs the survival of AML xenografted mice [93]. By contrast, CaMKI overexpression does not affect leukaemia development, but overexpression of the kinase-inactive CaMKI K49E mutant significantly decreases AML colony formation and increases mouse survival in AML transplantation studies [93], demonstrating the importance of CaMKI activity in controlling cell proliferation. Similar to expression of a dominant negative CaMKK mutant, expression of a dominant negative CaMKI mutant significantly decreases medulloblastoma cell migration [109], highlighting the importance of the CaMKK-CaMKI cascade in this cellular process. Murine CKLiK is upregulated by the haematopoietic cell-specific ETS family transcription factor, PU.1, in murine erythroleukemia cells, and is involved in apoptosis [122], suggesting that the human homolog of CKLiK may function in a similar fashion. These studies demonstrate that CaMKI can control cell proliferation, migration, and survival in medulloblastoma, breast and haematopoietic cancers, and therefore, may be a potential anti-cancer target for these cancer types.

### 3.3. CaMKII

Tumour cells express an entirely different spectrum of CaMKII isozymes than adult neuronal tissue or their non-transformed tissue counterparts. Eight distinct β, γ and δ CaMKII isozymes have been identified in human mammary tumour and neuroblastoma cell lines [123]. In addition to expression of these novel tumour variants, CaMKII is also overexpressed in a variety of cancer types (Table 1). CaMKII is more highly expressed in stage III and IV endometrial carcinomas when compared to stage I and II, and is associated with PCNA-labelling, clinical state, histological grade, and the presence of invasion, indicating that similar to CaMKI, CaMKII may play a role in endometrial carcinoma progression [94]. CaMKII is overexpressed in colon cancer compared to adjacent normal tissue and increases with poor differentiation [99], and in primary breast cancer compared to adjacent normal breast, as well as in lymph node metastases [100], indicating that CaMKII may play a role in cancer progression. In regards to specific isoforms, CaMKIIγ is increased in AML patient blasts compared to normal peripheral blood cells [98], in lung cancer compared to normal lung tissue [103] and is upregulated at diagnosis in chronic myeloid leukaemia (CML) [97]. Additionally, CaMKIIγ expression is increased in CML blasts resistant to tyrosine kinase inhibitors [97], glioma cells resistant to the Fas agonistic antibody (CH-11) [124] and also in TRAIL-resistant melanoma cells [125], suggesting that it may play a role in chemoresistance. 

The single-nucleotide polymorphism (SNP) rs10023113 in *CAMK2D* is associated with poor survival of early-stage non-small cell lung cancer patients [104], and high expression of *CAMK2G* in lung cancers is associated with significantly worse overall survival in 3 different cohorts [103]. Additionally, breast cancer samples that express high levels of *CAMK2* exhibit significantly worse overall and distant metastasis-free survival compared to patients with low *CAMK2* expression [100]. These studies suggest that *CaMK2* genes are potential prognostic biomarkers for a range of cancer types.

Furthermore, not only is CaMKII expression increased in a variety of cancer types, enhanced autophosphorylation at T286 is also frequently observed (Table 1). Increased T286/7 phosphorylation of CaMKII is noted in TRAIL-resistant melanoma cells [125], lung cancer oncospheres [103], osteosarcoma [101,102], leukaemia stem cells [126], AML patient blasts [98], primary and metastatic breast tumours compared to normal adjacent breast tissue [100] and in metastatic gastric cancers compared to non-metastatic tissues [105], indicating that enhanced CaMKII activity is a feature of cancers, and is also associated with metastatic disease.

Each of the CaMKII isoforms has been implicated in the control of a variety of cancer-related functions. In vitro, CaMKII controls cellular differentiation in AML differentiation [98] and cell proliferation in smooth muscle cells [127,128,129,130], lung cancer [111], medullary thyroid cancer [112], AML [98], glioma [120], T cell lymphoma [116], osteosarcoma [101], and colon [99], gastric [113] and prostate cancers [114]. In vivo, CaMKII controls osteosarcoma [101,102,131] and T cell lymphoma [116] tumour growth. CaMKII is essential for metastatic processes, including cell migration and invasion in osteosarcoma [101,102,131], glioma [118,119,120], and gastric [105,113], colon [99], breast [100], and prostate cancers [114,115]. CaMKII activity is essential for this process, as expression of a constitutively active (H282R) mutant enhances gastric cancer cell migration and invasion by upregulating matrix metalloproteinase-9 (MMP-9) [105]. 

Furthermore, CaMKIIγ has been implicated in tumourigenesis in a variety of cancer types. CaMKIIγ deletion suppresses T cell lymphomagenesis in vivo [116]. CaMKIIγ knockout mice develop fewer tumours, that are smaller than their wild-type counterparts, in a dextran sodium sulfate (DSS) and azoxymethane (AOM) model of colitis-associated tumourigenesis. Furthermore, only knockout in colonic tissue-resident cells, and not in bone marrow-derived immune cells, is involved in this suppressive effect [132]. By contrast, CaMKIIγ overexpression increases colon proliferation rates, decreases cell death and increases distal colitis-associated cancer compared to control mice [132]. CaMKIIγ^−/−^ mice exhibit increased tumour number and volume in diethylnitrosamine (DEN) and DEN followed by tumour promotor models of hepatic cancer [133]. Additionally, CaMKIIγ knockdown inhibits lung cancer tumourigenicity, and overexpression enhances tumourigenicity in vitro and in vivo [103].

Differentially phosphorylated CaMKII also controls different cellular functions. For example, dephosphorylation of CaMKII at T253 controls the metaphase-anaphase transition in neuroblastoma (SHSY5Y) and breast cancer (MDA-MB-231) cell lines [117]. Overexpression of wild-type or a T286 phospho-mimic mutant (T286D) of CaMKIIα increases cancer cell proliferation [117], migration and invasion [100]. By contrast, overexpression of a T253 phospho-mimic mutant (T253D) prevents cancer cell proliferation [117]. Taken together, these studies highlight the importance of CaMKII in controlling cancer cell proliferation and metastatic processes in a range of cancer types, indicating that its role in these functions is not cell-type specific but broadly applicable, and indicates that CaMKII may be a valid anti-cancer target for a variety of cancer types.

### 3.4. CaMKIV

Despite the restricted expression of CaMKIV in normal tissues, CaMKIV is overexpressed in several different types of cancer (Table 1). CaMKIV expression and activity is increased in hepatocellular carcinoma [106], small cell lung cancer [111], and is significantly associated with clinical stage, myometrial invasion and clinical outcome in endometrial carcinoma [134]. Furthermore, high *CAMK4* expression is associated with significantly worse overall survival for AML patients [93], and in endometrial carcinoma [135].

As CaMKIV is expressed in immune cells, it is not surprising that it has been shown to regulate haematopoietic stem cell homeostasis [16]. Additionally, CaMKIV has been implicated in cell proliferation and cell cycle regulation. Decreasing CaMKIV expression inhibits AML development in vitro and in vivo [93], and decreases hepatic cancer cell proliferation [86]. By contrast, overexpression of CaMKIV in AML cells arrests cells in G_0_/G_1_, in an activity dependent manner [121], suggesting that CaMKIV may have cell line dependent effects, even within the same cancer subtype.

## 4. The CaMK Family Are Potential Anti-Cancer Therapeutic Targets

Due to the importance of CaMK family members in controlling cancer-related functions, their suitability as anti-cancer targets have begun to be explored. Several pharmacological inhibitors that inhibit the activity of these enzymes have been developed, and their usefulness as anti-cancer treatments in a variety of cancer types has been examined.

### 4.1. STO-609

**Figure 5:**
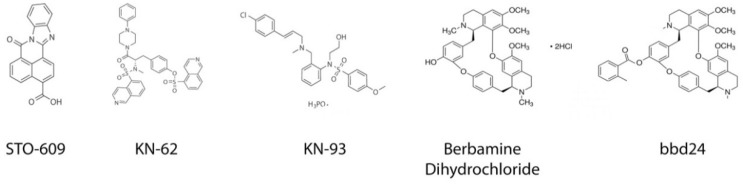
Structures of STO-609, KN-62, KN-93, berbamine dihydrochloride and bbd24.

7-Oxo-*7H*-benzimidazo[2,1-*a*]benz[de]isoquinoline-3-carboxylic acid (STO-609, Figure 5) is a selective CaMKK antagonist that inhibits autophosphorylation of both CaMKKα and CaMKKβ [136], without any significant effect on CaMKI and CaMKIV. However, at doses ~100-fold higher than the half maximal inhibitory concentration (IC_50_) for CaMKK, CaMKII and myosin light chain kinase (MLCK) are also inhibited. Additionally, STO-609 has been demonstrated to bind to and activate the aryl hydrocarbon receptor (AhR) [137], indicating that STO-609 may not be as CaMKK selective as initially believed. As CaMKK controls cancer cell proliferation, migration and survival in a variety of cancer types in vitro (Table 2), inhibiting CaMKK activity may be a valid anti-cancer therapeutic strategy in these cancer types.

Indeed, STO-609 decreases AML [93], prostate [89,91], gastric [108], hepatocellular [86] and ovarian cancer cell proliferation [88] in vitro, and induces apoptosis in ovarian [88] and gastric cancer lines [108]. Furthermore, treatment with STO-609 significantly reduces tumour burden in prostate and hepatocellular cancer mouse models in vivo (Table 3). Systemically administered STO-609 decreases tumour growth, both as a single agent and additively in combination with AR inhibition, in a C4-2B prostate cancer xenograft model [91], and in the DEN-induced hepatocellular carcinoma mouse model [86], demonstrating that CaMKK inhibition is a valid strategy for the treatment of prostate and hepatic cancer, and based on the in vitro studies, may potentially be suitable for other cancer types, including AML, gastric and ovarian cancers.

Intraperitoneal doses of STO-609 up to 300 µM/kg, or daily injections of 30 µM/kg for 4 weeks, have been shown to be well-tolerated in C57Bl/6 J mice, and did not induce parameters of liver or kidney toxicity [138]. However, the primary limitation of the use of STO-609 as an anti-cancer treatment is its poor solubility, therefore improved derivatives that increase solubility, whilst increasing efficacy will need to be developed.

### 4.2. KN-62/KN-93

4-[(2*S*)-2-[(5-Isoquinolinylsulfonyl)methylamino]-3-oxo-3-(4-pheynl-1-piperazinyl)propyl] phenylisoquinolinesulfonic acid ester (KN-62) and *N*-[2-[[[3-(4′-chlorophenyl)-2-propenyl]-methylamino]methyl]phenyl]-*N*-(2-hydroxyethyl)-4′-methoxybenzenesulfonamide phosphate salt (KN-93) (Figure 5), are membrane permeable CaMKII inhibitors that are competitive for CaM [140]. They were originally described as CaMKII specific inhibitors, as they do not affect other CaM-dependent enzymes, such as AMPK and MLCK [141].

However, subsequent studies have shown that they also inhibit CaMKI and CaMKIV and other molecules unrelated to the CaMK family, including ion channels [142,143,144,145,146]. Both KN-62 and KN-93 prevent the activation of CaMKII, but do not inhibit autonomously active CaMKII [142]. As CaMKI, CaMKII and CaMKIV control cancer cell proliferation, migration and survival in a variety of cancer types in vitro (Table 2), inhibiting the activity of these kinases using KN-62 and KN-93 may be a valid anti-cancer therapeutic strategy for a range of cancer types.

Indeed, KN-93 decreases proliferation in osteosarcoma [131], AML [98], T cell lymphoma [116], prostate cancer [114], endometrial cancer [94], glioma [120], colon cancer [99], breast cancer [110] and medullary thyroid cancer cells [112], induces apoptosis in prostate cancer [147,148], but not AML cells [98], and resensitises resistant melanoma cells to TRAIL-induced apoptosis [125] and resistant glioma cells to CH-11 [124,149] in vitro. Whilst KN-62 treatment does not result in apoptosis in cancer cell lines, it induces differentiation in AML cell lines [150], suppresses hypoxia inducible factor (HIF)-1α in hepatoma cells [151], potentiates the effects of etoposide in head and neck squamous cell carcinoma [152] and AML cell lines [153], and reverses adriamycin resistance in human ovarian cancer cells [154], indicating that it may be useful when combined with additional therapies. Additionally, KN-93 decreases migration and invasion in osteosarcoma [131], breast [100], prostate [114], colon [99] and endometrial [94] cancer cells. Similar findings have been observed with KN-62, as it inhibits gastric cancer cell proliferation, invasion and migration in vitro [105]. Taken together, these studies indicate that inhibiting CaMK family members using KN-93 and/or KN-62 may be suitable for the treatment of metastatic cancer in a range of solid tumours.

Furthermore, KN-93 decreases tumour burden in osteosarcoma xenograft models in vivo (Table 3). Systemically administered KN-93 decreases tumour growth in subcutaneous and orthotopic (intratibial) MG-63 osteosarcoma models in vivo, both alone [102] and in combination with CBO-P11 (a vascular endothelial growth factor receptor (VEGFR) inhibitor) [131].

Whilst in vivo studies using these inhibitors do not describe any side-effects, KN-62 and KN-93 have been widely used to examine a variety of heart and brain-related functions. For example, KN-62 and KN-93 can depress the rate of beating of cultured myocytes [155] and afterdepolarisation in the heart [156], respectively, and can block long-term potentiation (LTP) in rat hippocampus [157], indicating that they are likely to affect cardiac, learning and memory processes. Due to these potential side effects, as well as the range of off-target effects using these inhibitors, these inhibitors are unlikely to be translated into clinical use without modification or the use of alternative more cancer-selective modes of drug delivery (e.g., nanoparticles or liposomes).

### 4.3. Substrate Based Inhibitors: Autocamtide-3 Derived Peptide Inhibitor (AC3-I) and Autocamtide-2-Related Inhibitory Peptide (AIP)

Long inhibitory peptides based on the autoinhibitory domain of CaMKIIα have been developed. The N-terminus of this peptide contains the autophosphorylation site forming the basis for peptide substrates such as autocamtide-2 and -3 [158] and the development of a non-phosphorylatable analogue of autocamtide-2 generated the peptide inhibitors AIP (KKALRRQEAVDAL) [140] and AC3-I (KKALHRQEAVDCL) [159]. AIP competes with the active site of CaMKII, inhibiting activity regardless of how CaMKII was activated, and inhibits CaMKII with over 100-fold selectivity relative to protein kinase C (PKC), PKA and CaMKIV. However, altered selectivity can also occur when peptides are fused to GFP or modified by lipids to increase membrane permeability. For example, myristoylated AIP has been shown to have effects unrelated to CaMKII inhibition [160], indicating the presence of an additional non-CaMKII-related target, and green fluorescent protein (GFP)-AC3-I can inhibit cellular actions of protein kinase D1 (PKD1) [161]. AIP significantly attenuates glioma [119,162] and breast cancer [100] migration and invasion. Furthermore, AC3-I treatment arrests ovarian carcinoma cells in G_2_ and stops proliferation [163]. These studies indicate that CaMKII inhibition may be a viable therapeutic option for the treatment of metastatic disease.

### 4.4. CaMKIIN Derived Peptides (CaMKIINtide)

Small endogenous proteins that inhibit CaMKII (CaMKIIN) have been identified in rat brain [164] and there are two known rat isoforms—CaMKIINα [164] and CaMKIINβ [165]. Human CaMKIIN has also been identified in human dendritic cells [166]. CaMKIIN is implicated in the control of progression of cells through S phase. Overexpression of hCaMKIIN in colon cancer cells decreases cell proliferation, whereas silencing increases cell growth rates [166,167]. Furthermore, hCaMKIIN overexpression in ovarian cancer cells decreases proliferation and tumourigenicity [168,169], and also reduces migration and colony formation [169]. This implies a potential application of hCaMKIIN in the treatment of colon and ovarian cancers.

Identification of the core inhibitory domain of CaMKIIN led to the generation of a 28 amino acid peptide inhibitor called CaMKIINtide [164]. CaMKIINtide only inhibits activated CaMKII [164], and does not inhibit PKC, PKA, CaMKI, CaMKIV or CaMKK [164]. CaMKIINtide has also been modified to increase potency [170,171]. A cell permeable form, antCaNtide, decreases medullary thyroid cancer [112], and AML cell proliferation [121] indicating that the development of additional CaMKII specific inhibitors may provide viable therapeutic options for the treatment of haematological and thyroid cancers.

### 4.5. Berbamine Dihydrochloride

Berbamine (Figure 5) is a natural bis-benzylisoquinoline alkaloid, isolated from the traditional Chinese herbal medicine *Berberis amurensis*. Berbamine exhibits potent antitumour activities with low toxicity in a variety of cancer types, including melanoma, hepatocellular carcinoma, breast cancer, leukaemia and lung cancer [126,172,173,174,175,176,177,178]. Recently, berbamine was shown to produce its anti-cancer effects by blocking the ATP binding pocket of CaMKIIγ [126], however, berbamine also inhibits molecules unrelated to CaMKII, including mechano-electrical transducer channels, the Bcr/Abl fusion gene, and the NF-κB pathway [175,179,180]. 2-methylbenzoyl berbamine (bbd24) (Figure 5), a derivative of berbamine and a more potent CaMKII inhibitor, has been identified [126]. Berbamine inhibits the growth and reduces the viability of liver cancer [139] and CML cells [126] in a CaMKIIγ dependent manner. Bbd24 also kills liver cancer cells in vitro [139].

Berbamine has been shown to reduce tumour burden in several different animal models (Table 3). Berbamine decreases liver cancer [139] and CML [126] tumour burden in vivo and decreases tumour burden and significantly increases survival in an *N*-methyl-*N*-nitrosurea (MNU)-induced lymphoma model [116].

The main limitation for the use of berbamine clinically as an anti-cancer agent is its short plasma half-life and poor bioavailability at the tumour site after systemic administration. To circumvent this, lipid-based nanoparticles loaded with berbamine have been developed and have been shown to decrease primary tumour growth in a B16F10 mouse melanoma model and also suppress the incidence of lung metastases in vivo [181]. This highlights that newer mechanisms of drug delivery may be useful clinically to increase the cancer-specificity of these drugs, without enhancing toxicity.

## 5. Concluding Remarks and Perspectives

The CaMK family members, particularly CaMKII, are attractive anti-cancer targets as they are overexpressed in a plethora of cancer types, compared to adjacent normal tissue, and are vital in the modulation of cancer cell proliferation, survival, invasion and migration. Several targeted and broad-acting CaMK inhibitors have demonstrated pre-clinical anti-cancer efficacy in vivo. Many of the previously believed ‘CaMK-specific’ inhibitors have been shown to have a variety of off-target effects, which further limits their clinical usefulness. By contrast, CaMKIIN-tide, and its modified derivatives, based on the endogenous CaMKII inhibitory protein (CaMKIIN) have no described off-target effects, and are the most promising lead compounds for further development described here-in, however, their usefulness in vivo remains to be investigated. Additionally, as the CaMK family members are also involved in highly important, non-cancer related functions, direct inhibition using these existing CaMK inhibitors are likely to produce a range of deleterious side-effects if used clinically. Therefore, to be useful therapeutically, cancer-specific inhibitors or more cancer-specific modes of drug delivery would be required to be developed. One such potential strategy would be to encapsulate these CaMK inhibitors in nanoparticles or liposomes that specifically target cancer cells for the delivery of the inhibitor. Whilst this has been examined in other disease models for CaMKIIN inhibitory peptides, such as heart disease and asthma [182,183], they remain to be tested in cancer.

## Figures and Tables

**Table 1:** The Ca^2+^/calmodulin-stimulated protein kinase (CaMK) family is overexpressed in a range of cancer types. AML acute myeloid leukaemia; BPH Benign prostatic hyperplasia; ccRCC clear cell renal cell carcinoma; CML chronic myeloid leukaemia; GBM glioblastoma multiforme; GOBO gene expression based outcome for breast cancer; GWAS Genome Wide Association Study; HBV Hepatitis B virus; HCC hepatocellular carcinoma; NHT Neoadjuvant hormone therapy; NSCLC non-small cell lung cancer; PIN Prostate intraepithelial neoplasia; TCGA the cancer genome atlas; TMA tissue microarray.

CaMK Family Member	Cancer	Sample Type	Expression	Reference
CaMKKβ	Prostate	Prostate cancer TMA (*n* = 84); NHT TMA with hormone naïve, NHT <3 months, 3–6 months, or >6 months, or castrate-resistant (*n* = 107)	Increased protein expression in prostate cancer compared to PIN and BPH and in castrate-resistant cancer. Reduced expression following NHT.	[91]
		Prostate cancer progression (*n* = 5)	Increased protein expression in prostate cancer compared to normal prostate and with increasing Gleason score	[89]
		Normal prostate and prostate cancer TMA (*n* = 80), cancer, adjacent normal and metastases TMA (*n* = 95)	Increased protein expression in primary prostate cancer and bone metastasis compared to normal prostate	[90]
	Gastric	Gastric adenocarcinoma and normal oesophagus TMA (*n* = 98)	Increased protein expression in gastric tumours compared to normal oesophagus	[85]
	Liver	Hepatocellular carcinoma transcriptome profile microarray (*n* = 247); matched normal and tumour (*n* = 22).	Increased expression in liver cancer and *CAMKK2*^high^ correlates with poor disease-free survival. CaMKKβ protein upregulated in tumour compared to adjacent normal tissue.	[86]
	Glioma	Human glioma and normal brain tissue (*n* = 147 for expression and n = 101 for methylation); Whole genome mRNA expression microarray (*n* = 305 diffuse glioma samples, *n* = 151 methylation microarray, *n* = 275 GBM)	*CAMKK2* mRNA and protein is more highly expressed in high-grade gliomas compared to low-grade. Increased expression and *CAMKK2*^high^ correlates with poor overall survival. *CAMKK2* is differentially methylated between low and high grade glioma.	[87]
	Ovarian	High grade serous papillary ovarian cystadenocarcinoma and high-grade ovarian carcinoma with mucinous features (*n* = 4)	Increased protein expression in high grade serous papillary cystadenocarcinoma and high-grade ovarian cancer with mucinous features compared to non-malignant stromal tissue.	[88]
CaMKI	AML	TCGA AML database (*n* = 186)	*CAMK1D*^high^ correlates with poor overall survival	[93]
	Endometrial cancer	Endometrial carcinoma and normal endometria (*n* = 31 and *n* = 20)	Protein expression is associated with PCNA-labeling, stage, histological grade, the presence of invasion and outcome	[94]
	Breast cancer	Primary breast ductal carcinoma (*n* = 35)	*PNCK* mRNA is more highly expressed in a subset (8/23) of human breast tumours compared to benign breast tissue	[95]
	ccRCC	ccRCC and adjacent normal tissue (*n* = 92) and primary ccRCC tissue (*n*-248)	*PNCK* mRNA and protein expression higher in tumour compared to normal. Patients with *PNCK*^high^ have shorter overall survival	[96]
CaMKII	CML	Peripheral blood (*n* = 15 at diagnosis; *n* = 30 in chronic phase with remission; *n* = 26 in chronic phase treatment-resistant; *n* = 30 in advanced phase; *n* = 20 healthy)	CaMKIIγ upregulated at diagnosis and in treatment resistance	[97]
	AML	Peripheral blood samples (*n* = 16)	Total and phosphorylation of CaMKIIγ at T287 increased in AML	[98]
	Endometrial cancer	Endometrial carcinoma and normal endometria (*n* = 31 and *n* = 20)	Protein expression is associated with PCNA-labeling, stage, histological grade, the presence of invasion and outcome	[94]
	Colon cancer	Paracancerous tissue, well-differentiated and poorly differentiated colon cancer (*n* = 5, *n* = 6, *n* = 6)	CaMKII protein expression increased in colon cancer compared to paracancerous tissue, and increased with poor differentiation	[99]
	Breast cancer	GOBO Breast Cancer Database (*n* = 1881); Normal, primary and metastatic breast cancer TMA (*n* = 40, *n* = 70, and *n* = 10)	*CAMK2*^high^ associated with worse overall and distant metastasis free survival. Total CaMKII protein and T286/7 phosphorylation is increased in primary breast cancer and metastases	[100]
	Osteosarcoma	Chondroblastic, osteoblastic and fibroblastic subtypes (*n* = 114)	Phosphorylation of αCaMKII at T286 is increased in osteosarcoma compared to normal osteoblasts and mesenchymal stromal cells	[101]
		Primary osteosarcoma tumours (*n* = 4)	Phosphorylation of αCaMKII at T286 is increased in osteosarcoma	[102]
	Lung cancer	Oncomine databases (*n* = 187, *n* = 226, *n* = 130)	*CAMK2G*^high^ associated with worse overall survival	[103]
		GWAS in NSCLC patients (*n* = 354)	Rs10023113 in *CAMK2D* associated with survival	[104]
	Gastric cancer	Non-metastatic and metastatic gastric cancer tissues (*n* = 10, and *n* = 10)	Phosphorylation at T286 is increased in metastatic compared to non-metastatic tissue	[105]
CaMKIV	AML	TCGA AML database (*n* = 186)	*CAMK4*^high^ correlates with poor overall survival	[93]
	HCC	Normal liver, chronic hepatitis, cirrhosis, and HCC (*n* = 4, *n* = 6, *n* = 4, *n* = 12)	CaMKIV protein expression and activation increased in HCC compared to normal liver and cirrhosis	[106]

**Table 2:** The calcium/calmodulin-stimulated protein kinase family mediate a variety of cancer-related functions in multiple cancer types in vitro. AML acute myeloid leukaemia; AIP autocamtide-2 inhibitory peptide; HCC hepatocellular carcinoma; siRNA small interfering RNA; shRNA short hairpin RNA; WT wild-type.

Target	Cancer	Cell Line(s)	Method of Manipulation	Effect	Reference
CaMKK	Prostate	LNCaP	Pharmacological inhibition (STO-609)	Decreased proliferation	[89]
		LNCaP, VCqP, C4-2B, 22Rv1	siRNA and pharmacological inhibition (STO-609)	Decreased proliferation	[91]
		LNCaP	siRNA and pharmacological inhibition (STO-609)	Decreased migration and invasion	[92]
		LNCaP	CaMKKβ overexpression	Increased migration	[92]
		LNCaP	CaMKKβ overexpression	Decreased proliferation	[90]
		DU145	CaMKKβ siRNA	Decreased proliferation	[107]
	Gastric	AGS, KATO-III, SNU-16, N87	CaMKKβ siRNA	Decreased proliferation	[85]
		SNU-1, N87	CaMKKβ siRNA and pharmacological inhibition (STO-609)	Decreased proliferation and induced apoptosis	[108]
	HCC	PHM1, SK-Hep1, HepG2	CaMKKβ siRNA and pharmacological inhibition (STO-609)	Decreased proliferation	[86]
	Glioma	U-87MG	CaMKKβ siRNA	Decreased proliferation, migration and invasion	[87]
	Ovarian	SKOV-3, OVCAR-3	CaMKKβ siRNA and pharmacological inhibition (STO-609)	Decreased proliferation and induced apoptosis	[88]
	Breast cancer	MCF-7	CaMKKα and CaMKKβ siRNA	Arrested cells in G_1_	[110]
	Medulloblastoma	DOAY	Expression of dominant negative CaMKK mutant	Decreased migration	[109]
CaMKI	AML	MV-4-11, Kasumi-1	shRNA and CaMKI overexpression	Downregulation decreased proliferation; Overexpression of kinase dead mutant decreased colony formation	[93]
	Breast cancer	MCF-7	siRNA	Arrested cells in G_1_	[110]
	Medulloblastoma	DOAY	Expression of dominant negative CaMKI mutant	Decreased migration	[109]
CaMKII	Osteosarcoma	MG-63, 143B, HOS	CaMKIIα shRNA and overexpression	Knockdown decreased proliferation, migration and invasion. Overexpression increased proliferation, migration, invasion	[101]
		MG-63, 154B	Wild-type and K42M kinase dead CaMKIIα overexpression	K42M kinase dead overexpression reduced growth	[102]
	AML	KG1, KCL22, THP-1, Kasumi-1	Overexpression of kinase dead truncated CaMKIIγ, CaMKIIγ shRNA, pharmacological inhibition (KN-62, KN-93, KN-92)	Kinase dead overexpression, shRNA and pharmacological inhibition decreased colony formation and proliferation.	[98]
	Lung cancer	SCC-9, NCI-H345, NCI-H128, NCI-H146, NCI-H69	Pharmacological inhibition (KN-62)	Slowed progression through S phase and decreased proliferation	[111]
	Medullary thyroid cancer	TT, MZ-CRC1	Pharmacological inhibition (antCaNtide)	Decreased cell proliferation	[112]
	Colon cancer	HCT116	Pharmacological inhibition (KN-92, KN-93)	Decreased proliferation, migration and invasion	[99]
	Gastric cancer	BGC-823	Pharmacological inhibition (KN-93) and CaMKIIβ shRNA	Decreased cell proliferation and migration, induced apoptosis	[113]
		BGC-823	Pharmacological inhibition (KN-62) and H282R constitutively active CaMKIIα overexpression	Pharmacological inhibition decreased cell proliferation. Overexpression of constitutively active increased cell proliferation, migration and invasion	[105]
	Prostate cancer	C4-2B, LNCaP, PC3, DU145	Pharmacological inhibition (KN-93)	Decreased proliferation	[114]
		1542-CP3TX	Pharmacological inhibition (AIP)	Decreased cell migration	[115]
	T cell lymphoma	H9	CaMKIIγ knockout by CRISPR/Cas	Decreased proliferation and colony formation	[116]
	Breast cancer	MDA-MB-231, MCF-7	Overexpression of CaMKIIα, T286D (phosphomimic) and T286V (phosphonull), Pharmacological inhibition (KN-92, KN-93, AIP)	Overexpression of WT and phosphomimic forms increased cell proliferation, migration and invasion. Pharmacological inhibition decreased migration and invasion	[100]
		MDA-MB-231	Overexpression of CaMKIIα, T286D (phosphomimic) and T253D (phosphomimic)	Overexpression of WT and T286D increased proliferation. Overexpression of T253D decreased proliferation	[117]
	Glioma	C6, U-251MG	Pharmacological inhibition (KN-93)	Decreased migration	[118]
		D54, H8a	Pharmacological inhibition (AIP)	Decreased migration	[119]
		U-87MG	CaMKIIγ siRNA, pharmacological inhibition (KN-93)	Decreased proliferation, invasion and neurosphere formation	[120]
CaMKIV	AML	Lin^−^ AML, MV-4-11, Kasumi-1	CaMKIV and K75M overexpression and CaMKIV shRNA	CaMKIV-K75M overexpression and shRNA knockdown decreased colony formation. shRNA knockdown induced apoptosis and decreased proliferation.	[93]
		U937	CaMKIV wild-type and K71M kinase dead mutant overexpression	Cells arrested in G_0_/G_1_ following WT, but not K71M, overexpression	[121]
	HCC	PHM1, SK-Hep1	CaMKIV siRNA	Inhibited colony formation and proliferation	[86]

**Table 3:** Effects of pharmacological inhibitors of CaMK family members on tumour burden in in vivo animal models of cancer. AR androgen receptor; CML chronic myeloid leukaemia; DEN diethylnitrosamine; HCC hepatocellular carcinoma; MNU *N*-methyl-*N*-nitrosourea; NOD-SCID non-obese diabetic severe combined immunodeficient; NSG nod scid gamma.

Pharmacological Agent	Cancer	Model	Treatment Schedule	Outcome	Reference
STO-609	Prostate	Subcutaneous C4-2B xenograft in full and castrated nude mice	10 µmol/kg STO-609 or vehicle intraperitoneally three times/week	Reduction in tumour growth, which was enhanced in castrated mice	[91]
	HCC	DEN-induced hepatic cancer model	30 µg/kg STO-609 or vehicle intraperitoneally twice/week for 4 weeks	Reduction in tumour growth	[86]
KN-93	Osteosarcoma	Subcutaneous and intratibial MG-63 xenograft in nude mice	1 mg/kg saline or KN-93 intraperitoneally every other day for 6 weeks	Reduction in tumour growth	[102]
		Intratibial 143B xenograft in nude mice	Osmotic pump delivery of 5 µg/µL KN-93, 10 µg/µL CBO-P11 or vehicle set to release 0.25 µL/h for 2 weeks	Reduction in tumour growth alone and in combination with CBO-P11	[131]
Berbamine	HCC	Subcutaneous Huh7 or SK-Hep-1 xenograft in NOD-SCID mice	100 mg/kg berbamine orally twice day for 5 consecutive days, 2 days withdrawal, and then repeated once	Reduction in tumour growth	[139]
	CML	Subcutaneous K562 and primary CML cells from a patient at blast crisis xenograft in nude mice	100 mg/kg berbamine, imatinib or vehicle orally three time daily for 10 days	Reduction in tumour growth	[126]
	T cell lymphoma	MNU-induced lymphoma model and subcutaneous H9 xenograft in NSG mice	50 m 100 or 150 mg/kg berbamine, or vehicle, orally administered to mice 2 times a day for 14 days, 14 days withdrawal, cycle repeated; Xenograft study: 150 mg/kg berbamine or vehicle oral twice a day	Reduction in tumour growth in both models	[116]
